# Wood‐Derived Carbon Fibers Embedded with SnO*_x_* Nanoparticles as Anode Material for Lithium‐Ion Batteries

**DOI:** 10.1002/gch2.201900048

**Published:** 2019-11-08

**Authors:** Janardhanan Revathi, Adduru Jyothirmayi, Tata Narasinga Rao, Atul Suresh Deshpande

**Affiliations:** ^1^ Centre for Nano Materials International Advanced Research Centre for Powder Metallurgy and New Materials Balapur Hyderabad Telangana 500005 India; ^2^ Department of Materials Science and Metallurgical Engineering IIT Hyderabad Kandi Hyderabad Telangana 502285 India

**Keywords:** anode materials, carbon–tin composites, lithium‐ion batteries, tin oxide, wood fibers

## Abstract

Carbon–SnO*_x_* composites are obtained by impregnating acetylacetone‐treated, delignified wood fibers with tin precursor and successively carbonizing at 1000 °C in 95% argon and 5% oxygen. Scanning electron microscopy and nitrogen sorption studies (Brunauer–Emmett–Teller) show that acetylacetone treatment stabilizes the wood fiber structure during carbonization at 1000 °C and preserves the porous structural features. X‐ray diffraction, transmission electron microscopy, and X‐ray photoelectron spectroscopy studies show that the small amount of oxygen introduced in inert atmosphere passivates the surface of tin nanoparticles. The passivation process yields thermally and electrochemically stable SnO*_x_* particles embedded in carbon matrix. The resultant carbon–SnO*_x_* material with 16 wt% SnO*_x_* shows excellent electrochemical performance of rate capability from 0.1 to 10 A g^−1^ and cycling stability for 1000 cycles with Li‐ion storage capacity of 280 mAh g^−1^ at a current density of 10 A g^−1^. The remarkable electrochemical performance of wood‐derived carbon–SnO*_x_* composite is attributed to the reproduction of structural featured wood fibers to nanoscale in carbon–SnO*_x_* composite and controlled passivation of tin nanoparticles to yield SnO*_x_* nanoparticles.

## Graphical abstract

Acetylacetone‐treated delignified wood fibers preserve the porous features of the parent wood even after carbonization. Such acetylacetone‐mediated synthesis of wood‐derived carbon fibers anchored with SnO*_x_* particles partially passivated during carbonization results in electrochemically stable carbon–SnO*_x_* composite. Wood‐derived carbon–SnO*_x_* composite due to its unique porous features displays excellent cycling stability and rate capability.

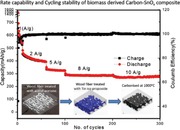

## 1 Introduction

Impetus for research in electrode materials for lithium‐ion batteries is driven by the increasing demand for energy storage devices having both high energy storage capacity and high out power for application such as electric vehicles (EVs). The commercial Li‐ion batteries show potential for high energy storage capacity. However, they still fall short of energy density required for EV applications. Additionally, at high charge/discharge current density, irreversible capacity loss due to destabilization of solid electrolyte interface (SEI) occurs. This combined with inherently slow electrochemical kinetics for lithiation/delithiation limits the use of Li‐ion batteries for high power density applications.^1^, ^2^, ^3^, ^4^ The electrochemical kinetics can be improved by employing nanosized electrode materials which offer low diffusion length and faster ionic mobility. However, structural integrity is lost during cycling that leads to unstable SEI layer and capacity fading.^5^ Nanostructured porous materials offer excellent structural integrity during cycling. Carefully engineered nanostructured porous carbon‐metal oxide composites contain inherent mechanical stability and a continuous electronic and ionic conduction network.^6^ Additionally, in situ preparation of these composites leads to strong interfacial interaction between carbon and metal oxides, which improves electrochemical contact. This also facilitates stable SEI layer formation, retains structural stability, and enhances electrochemical performance at higher charge/discharge rates.^2^, ^5^, ^6^, ^7^, ^8^, ^9^

Among anode materials, tin oxide‐based materials, i.e., SnO*_x_* (*x* = 0–2) are preferred for their low volume change (300%) when compared with Si (323%) and Ge (370%).^9^ Furthermore, SnO*_x_* (*x* = 0–2) overcomes unstable SEI layer in electrochemical alloying of Sn by lithium oxide formation. Nevertheless, capacity fading is observed in case of bulk SnO*_x_* materials.^9^

One way to overcome the limitations of SnO*_x_*‐based materials is by forming nanostructured SnO*_x_*–carbon composites. Various nanostructured SnO*_x_*–carbon composites such as Sn nanorods encapsulated in carbon,^10^ Sn/C composites Sn–C core–shell nanoparticles,^11^ carbon nanotube‐encapsulated Sn nanoparticles,^12^ etc., have been reported. Synthesis of these nanostructured SnO*_x_*–C with electrochemical conversion family of compounds has been achieved using various approaches. Typically, template assisted synthesis strategies using hard and soft template have been widely explored for the synthesis of nanostructured porous materials.^13^, ^14^, ^15^, ^16^, ^17^, ^18^, ^19^ Large‐scale production by using these methods are complex and expensive. In addition, extra steps are required for the removal of template such as chemical etching, calcination, etc. Thus, to avoid having additional steps, a nanostructured template that is also a carbon source would be ideal. Wood is a natural, porous composite material available in abundance. Natural wood has structure with various levels of hierarchy extending from macroscopic dimensions of stem up to nanoscopic dimensions of cellulose microfibrils. Individual wood cell or commonly known as wood fiber is a tubular structure with cell walls made up of multiple layers. Each layer consists of well‐aligned semicrystalline cellulose microfibrils with a diameter of about 2–4 nm embedded in a matrix of amorphous lignin and hemicellulose. Careful chemical treatment of wood allows selective removal of lignin and hemicellulose without significant change in cellulose microfibril organization to yield hierarchically porous cellulose matrix. This hierarchically porous matrix has been used as a template for deposition of nanoparticles to generate hierarchically porous ceramic materials.^20^

Apart from this, carbon material derived from wood pulp and composites derived from wood fibers have shown improved electrolyte wetting and holding capacity to achieve efficient ionic and electronic transfer in super capacitors and sodium‐ion batteries.^21^, ^22^, ^23^, ^24^ Even though there are reports on application of wood fiber for lithium‐ion batteries, use of wood fibers as carbon source as well as a template to exploit its structural features down to nanoscale is not reported so far.^22^, ^23^ In addition, thermal nonlinear dehydration of hydroxyl groups in cellulose decomposes the unique porous and structural features during carbonization. Acetylation and maleic anhydride functionalization of cellulose attempts to probe till nanoscale of cellulose microfibril has not served the purpose enough to retain the structure.^25^, ^26^ Thus, we believe that it is necessary to find an alternative chemical treatment of cellulose to control structural collapse. In this work, we have employed acetylacetone, a well‐known chelating agent for treatment of cellulose microfibrils in wood fibers. To obtain carbon–SnO*_x_* nanocomposite, delignified wood‐derived cellulose fibers treated with acetylacetone have been used as template. By careful impregnation of acetylacetone‐treated wood fibers with tin precursor, structure of wood fibers is replicated to a large extent in carbon–SnO*_x_* composite after carbonization. Carbonization is done at high heating ramp rate of 10 °C min^−1^ when compared with 2–5 °C min^−1^ reported in literature. In our opinion, slow heating rates give sufficient time for the structural features to collapse. Rapid heating rates prevent structural collapse and form stable porous carbon rapidly. We propose faster heating rates to address the nonlinear dehydration of cellulose during carbonization. To improve the electrochemical performance, unlike conventional carbonization for carbon–tin composites which is done in pure argon atmosphere, we introduced oxygen in inert atmosphere to passivate Sn nanoparticles. This in situ synthesis of carbon–SnO*_x_* composite results in electrochemically stable SnO*_x_*/passivated Sn particles embedded in carbon matrix. The cylindrical porous carbon matrix acts as a protective layer and mechanical buffer for SnO*_x_* particles and helps obtaining stable SEI layer in initial few cycles with minimal irreversible capacity loss.

## 2 Results and Discussion

**Figure 1:**
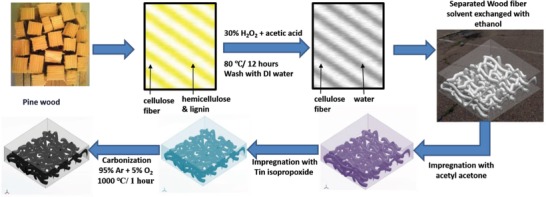
Schematic for synthesis of C–SnO*_x_* composite.

**Figure 2:**
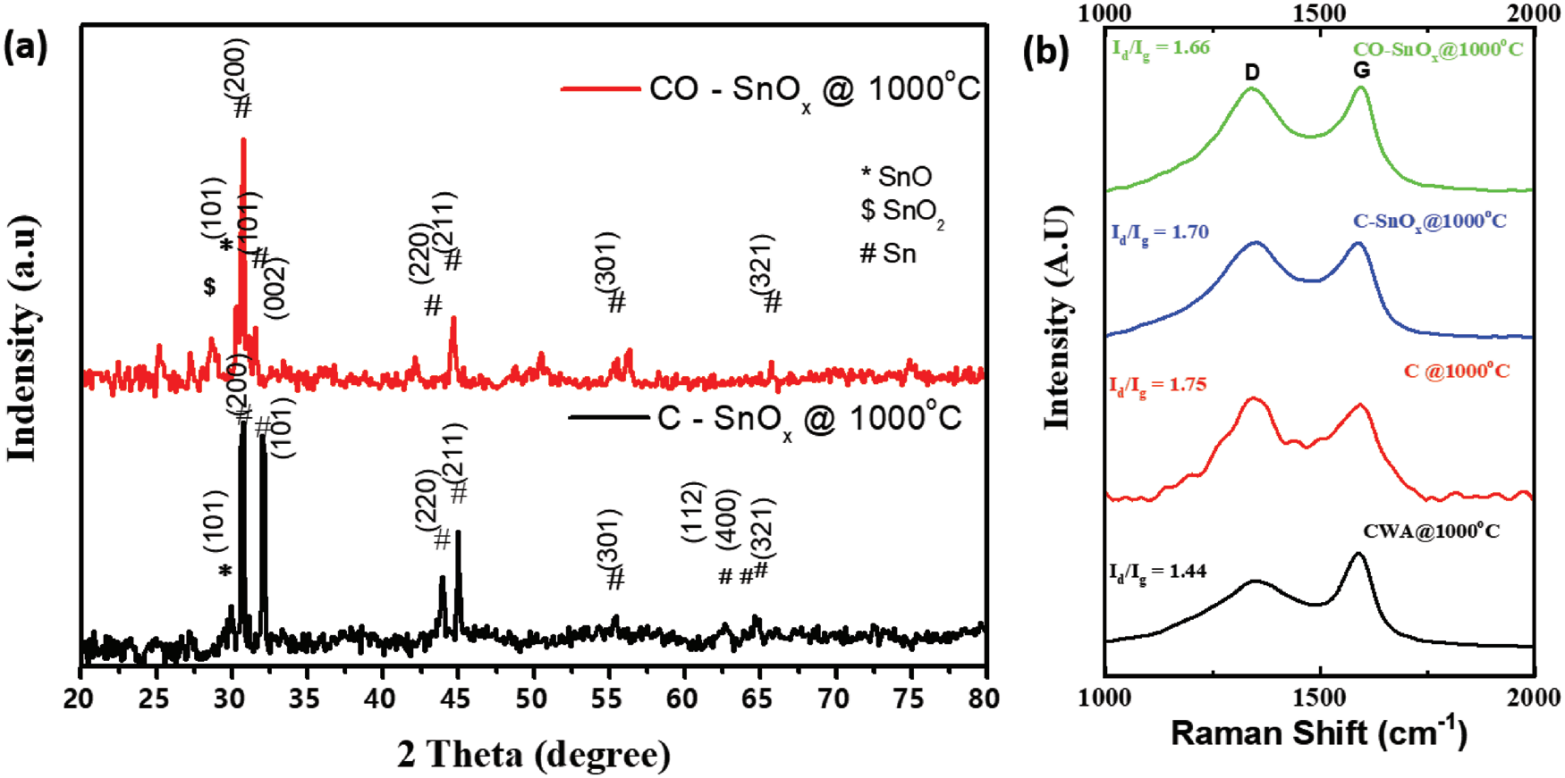
a) XRD of C–SnO*_x_* composite: i) CO–SnO*_x_*@1000 °C, ii) C–SnO*_x_*@1000 °C. b) Raman for i) CO–SnO*_x_*@1000 °C, ii) C–SnO*_x_*@1000 °C, iii) C@1000 °C, iv) CWA@1000 °C.

A porous C–SnO*_x_* composite was prepared by acetylacetone‐mediated templating of wood fibers with tin isopropoxide. Schematic illustration of fabrication of wood derived carbon fibre anchored with SnO*_x_* nanoparticles is depicted in **Figure** **1**. Briefly, wood fibers obtained by maceration of wood are solvent exchanged with ethanol three times followed by treating with acetylacetone. Then, tin isopropoxide in acetylacetone is added to wood fibers and dried at 80 °C for 12 h. Owing to fast hydrolysis of tin isopropoxide, acetylacetone was used as a chelating agent to delay the hydrolysis reaction. This ensures effective impregnation of tin isopropoxide into cellulose fiber matrix. Macerated wood fiber without further treatment, acetylacetone‐treated wood fiber, and tin isopropoxide impregnated wood fibers carbonized at 1000 °C in argon atmosphere were labeled as CWA@1000 °C, C@1000 °C, and C–SnO*_x_*@1000 °C, respectively. The tin isopropoxide impregnated wood fibers carbonized in argon atmosphere accompanied with 5% oxygen atmosphere introduced at the onset of 1000 °C is labeled as CO–SnO*_x_*@1000 °C. The X‐ray diffraction (XRD) pattern of C–SnO*_x_*@1000 °C (**Figure** **2**a) shows reflections corresponding to metallic tin (JCPDS file No. 96‐154‐0070). This indicates that carbonization at 1000 °C reduces tin precursor to metallic tin by carbothermal reduction to yield nanocrystalline Sn particles embedded in carbon matrix.^13^, ^14^ The XRD pattern for CO–SnO*_x_*@1000 °C in Figure 2a indicates the presence of metallic tin along with mixture of SnO (JCPDS file No. 96‐154‐901‐2141) and SnO_2_ (JCPDS file No. 96‐100‐0063). Thus, in case of CO–SnO*_x_*@1000 °C, presence of small amount of oxygen in carbonization atmospheres results in the partial oxidation of metallic tin. In addition, presence of oxygen may also lead to oxidative etching of disordered carbon, enhancing graphitic nature.^16^ To assess if any improvement in graphitic nature of carbon in CO–SnO*_x_*@1000 °C takes place, Raman spectra of CO–SnO*_x_*@1000 °C, C–SnO*_x_*@1000 °C, C@1000 °C, and CWA@1000 °C samples were compared (Figure 2b). All the samples show two characteristic Raman bands, namely, D band (≈1350 cm^−1^) associated with defects in graphitic layers and G band (≈1580 cm^−1^) corresponding to in plane vibrations of carbon atoms in graphite layers. The ratio of integrated intensities of D and G band, i.e., *I*_d_/*I*_g_ is taken as the measure of graphitic nature, i.e., smaller the value of *I*_d_/*I*_g_ ratio, better the graphitization. Comparison of *I*_d_/*I*_g_ ratio of all the samples (Table S1, Supporting Information) shows smallest *I*_d_/*I*_g_ ratio value for CO–SnO*_x_*@1000 °C sample which confirms the improved graphitization of carbon in CO–SnO*_x_*@1000 °C when compared with C–SnO*_x_*@1000 °C and C@1000 °C samples. CWA@1000 °C has lower *I*_d_/*I*_g_ ratio than C@1000 °C. This is due to uncontrolled rapid dehydration of cellulose in CWA@1000 °C compared to C@1000 °C that results in loss of porosity and better graphitization of carbon matrix.

**Figure 3:**
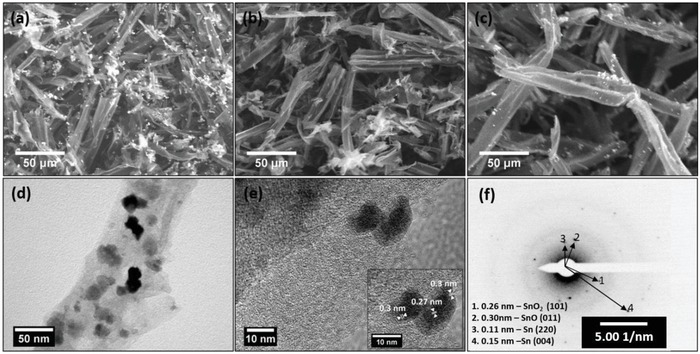
FESEM of a) C–SnO*_x_*@1000 °C, b) C@1000 °C, c) CO–SnO*_x_*@1000 °C. TEM for d,e) C–SnO*_x_*@1000 °C. Inset of (e) is the HRTEM of CO–SnO*_x_*@1000 °C. f) Selected area diffraction pattern for CO–SnO*_x_*@1000 °C.

**Figure 4:**
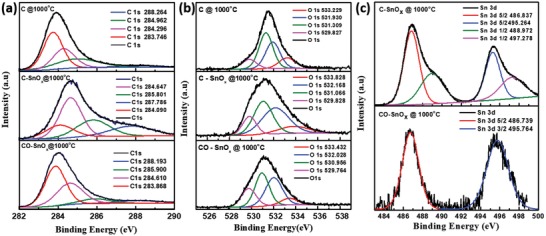
XPS for C@1000 °C and C–SnO*_x_* composites. a) C 1s spectra, b) O 1s spectra, and c) Sn 3d spectra.

The surface morphology of the carbonized samples was examined using field emission scanning electron microscopy (FESEM) analysis. As seen in **Figure** **3**b, the porous structural features of wood fibers were preserved after carbonization at 1000 °C in C@1000 °C. The individual fibers have tubular shape with average dimension of 30 µm diameter and 150 µm length. Absence of large lumps of precipitates confirms homogenous distribution of SnO*_x_* into carbon matrix in C–SnO*_x_*@1000 °C and CO–SnO*_x_*@1000 °C (Figure 3a,c). This is due to the presence of acetylacetone which acts as a chelating agent and delays the hydrolysis of tin isopropoxide and allows even impregnation of wood fibers. Apart from controlling the templating process, we also investigated the effect of acetylacetone treatment on morphology of delignified wood fiber during carbonization. Typically, delignification followed by drying leads to loss of porosity due to structural collapse. The carbonization of these fibers leads to further loss of structural features. To assess the effect of acetylacetone on morphology of resultant carbon, delignified wood fibers with and without acetylacetone treatment were carbonized at 1000 °C in an argon atmosphere. Figure S1 in the Supporting Information shows the FESEM of wood‐derived carbon fiber with acetylacetone treatment a) C@1000 °C and without acetylacetone treatment b) CWA@1000 °C. The inherent porous features of wood fiber are preserved even after carbonization with negligible shrinkage in acetylacetone‐treated delignified wood‐derived carbon fibers as shown in Figure S1a in the Supporting Information. In contrast, delignified wood‐derived carbon fiber without acetylacetone has undergone significant shrinkage with loss of porous features as seen in Figure S1b in the Supporting Information. Thus, it is evident that acetylacetone treatment helps in preserving the morphology of wood fibers during carbonization. However, the exact nature of interaction between cellulose fibers and acetylacetone and mechanism of structural stabilization need additional investigation and are beyond the scope of present work. The Brunauer–Emmett–Teller (BET) isotherm of CWA@1000 °C, C@1000 °C, C–SnO*_x_*@1000 °C, and CO–SnO*_x_*@1000 °C are shown in Figure S2 in the Supporting Information. The BET isotherms of C@1000 °C and CO–SnO*_x_*@1000 °C are of type IV isotherm with an irreversible hysteresis.^13^, ^17^, ^18^, ^19^ This reveals co‐presence of mesopores along with micropores (Table S2, Supporting Information). The BET isotherms confirm possibility for hierarchical arrangement of mesopores and micropores in carbon matrix originated from wood fiber template. This hierarchical arrangement is essential for efficient electrolyte wetting and establishes a continuous ionic and electronic conductivity network. BET surface area for CWA@1000 °C is low as mentioned in Table S2 in the Supporting Information when compared with its other three counterparts. The comparison of BET results and FESEM images (Figure 3a,c and Figure S1, Supporting Information) confirms that the acetylacetone‐treated wood‐derived carbon fibers have undergone negligible shrinkage with well‐defined porous features. Whereas wood‐derived carbon fibers have lost their porous features with enormous shrinkage. This clearly proves the ability of acetylacetone treatment of cellulose in hindering structural degradation of cellulose during carbonization. The results obtained by us are comparable with reports on acetylation and maleic anhydride surface functionalization of cellulose.^25^, ^26^ The presence of SnO*_x_* particles in C–SnO*_x_*@1000 °C and CO–SnO*_x_*@1000 °C contribute to their increase in surface area when compared with C@1000 °C. The increase in surface area of C–SnO*_x_*@1000 °C when compared with CO–SnO*_x_*@1000 °C sample may be due to oxidative etching of disordered carbon leading to increase in mesopores and fused meso and micropores in CO–SnO*_x_*@1000 °C sample that results in increased average pore size as shown in Table S2 in the Supporting Information. This is evident from low pressure segment of the BET isotherm of CO–SnO*_x_*@1000 °C. Thus, increase in average pore diameter results in lower surface area of CO–SnO*_x_*@1000 °C when compared with C–SnO*_x_*@1000 °C. The hierarchical porous structure observed in C–SnO*_x_*@1000 °C and CO–SnO*_x_*@1000 °C clearly indicates that templating of hierarchical porous structure of wood cells obtained after delignification took place down to nanometer level due to the use of acetylacetone which prevented hydrolysis of tin alkoxide leading to effective impregnation of wood cells. Further characterizations are done for all samples except CWA@1000 °C. The surface chemical compositions of C@1000 °C, C–SnO*_x_*@1000 °C, and CO–SnO*_x_*@1000 °C samples were investigated by X‐ray photoelectron spectroscopy (XPS), as shown in **Figure** **4**. From XPS survey spectra, it can be clearly observed that there are three peaks located around 284.8, 531.2, and 487.1 eV, which are assigned to C 1s, O 1s, and Sn 3d spectra as shown in Figure 4a–c, respectively. C 1s spectra of C@1000 °C sample are deconvoluted into four C 1s peaks at 283.7, 284.29, 284.96, and 288.26 eV as seen in Figure 4a. These peaks correspond to aromatic C=C, aromatic C–C, aliphatic C—C, and oxygen‐bonded O—C=O, respectively. In C–SnO*_x_*@1000 °C, C 1s peaks deconvolute into 284.09, 284.64, 285.8, and 287.78 eV, which correspond to aromatic C=C, aliphatic C—C, oxygen‐bonded C—O, and oxygen‐bonded C=O, respectively. In CO–SnO*_x_*@1000 °C, C 1s peaks deconvolute into 283.86, 284.6, 285.9, and 288.19 eV that correspond to aromatic C=C, aliphatic C—C, oxygen‐bonded C—O, and oxygen‐bonded C=O, respectively. Aromatic —C=C—, C 1s peak intensity is 16.52, 44.67, and 50.08 for C@1000 °C, CSnO*_x_*@1000 °C, and CO–SnO*_x_*@1000 °C, respectively. This confirms the presence of maximum graphitic carbon in CO—SnO*_x_*@1000 °C sample which is in compliance with Raman results.^27^ O 1s spectra for C@1000 °C, C–SnO*_x_*@1000 °C, and CO–SnO*_x_*@1000 °C are shown in Figure 4b. O 1s spectra deconvolute into 529.8, 531.3, 531.9, 533.2 for C@1000 °C, 529.8, 531.06, 532.16, 533.8 for C—SnO*_x_*@1000 °C, and 529.7, 530.95, 532.02, 533.43 for CO—SnO*_x_*@1000 °C. These peaks correspond to O^2−^ in MO*_x_*, chemisorbed H_2_O, organic C—O, and adsorbed oxygen, respectively. The presence of O^2−^ from MO*_x_* in C–1000 °C can be from traces of metal ions present in wood sample whereas in C—SnO*_x_*@1000 °C and CO–SnO*_x_*@1000 °C it is due to SnO*_x_*. Surface passivation of Sn nanoparticles in C–SnO*_x_*@1000 °C and CO–SnO*_x_*@1000 °C samples is confirmed by the presence of O^2−^ peak from tin oxide at 529.7 eV. This is in correlation with absence of metallic Sn peaks in Sn 3d spectra of same samples.^28^, ^29^, ^30^

Sn 3d spectra is shown in Figure 4c. In C–SnO*_x_*@1000 °C Sn 3d peaks deconvolute into four peaks 486.8, 495.2, 488.9, and 497.2 eV, which correspond to Sn 3d 5/2, Sn 3d 3/2, Sn 3d 1/2, and Sn 3d 1/2, respectively. Sn 3d peak of CO–SnO*_x_*@1000 °C deconvolutes into two peaks 486.7 and 495.7 eV that correspond to Sn 3d 5/2 and Sn 3d 3/2. All Sn 3d peaks present in C–SnO*_x_*@1000 °C and CO–SnO*_x_*@1000 °C are due to Sn^4+^ in SnO_2_. Absence of metallic Sn peaks confirms the surface passivation by SnO*_x_* formation on surface of Sn particles in C–SnO*_x_*@1000 °C sample. Also slight peak broadening observed in C–SnO*_x_*@1000 °C and CO–SnO*_x_*@1000 °C confirms co‐presence of SnO and SnO_2_. When compared with CO–SnO*_x_*@1000 °C sample, peak intensity for Sn 3d in C–SnO*_x_*@1000 °C is quite high. This confirms the presence of SnO*_x_* particles mostly on the surface in C–SnO*_x_*@1000 °C, whereas in CO–SnO*_x_*@1000 °C sample most of the SnO*_x_* is embedded in carbon matrix as seen in Figure 3a,c. A clear advantage of this is expected as superior electrochemical performance of CO–SnO*_x_*@1000 °C due to the surface passivation of SnO*_x_* embedded in carbon matrix.^28^, ^29^, ^30^

To evaluate the electrochemical performance, it is important to know that SnO*_x_* content is present in sample. Thermogravimetric analysis (TGA) is performed for CO–SnO*_x_*@1000 °C sample as shown in Figure S3 in the Supporting Information and the result of TGA revealed that SnO*_x_* content in sample is about 16 wt%. Onset of carbon burnout above 400 °C confirms partial crystallinity of carbon observed in Raman spectra. Electrochemical performance of C–SnO*_x_* composite anodes, namely, C–SnO*_x_*@1000 °C and CO–SnO*_x_*@1000 °C, for Li‐ion batteries is systematically investigated by using coin cells. For comparison, C@1000 °C prepared using acetylacetone‐treated wood fibers are also tested by using coin cells.

**Figure 5:**
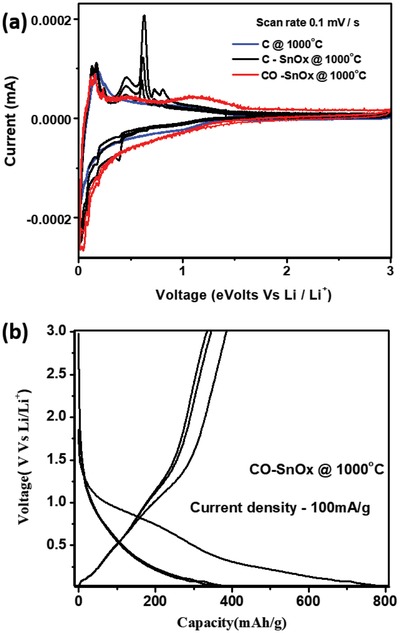
a) CV for C@1000 °C, C–SnO*_x_*@1000 °C, CO–SnO*_x_*@1000 °C. b) Capacity versus voltage profile for CO–SnO*_x_*@1000 °C at 100 mA g^−1^ current density.

To understand electrochemical lithiation/delithiation reactions of C@1000 °C, C–SnO*_x_*@1000 °C, CO–SnO*_x_*@1000 °C, cyclic voltammetry (CV) is performed at a scan rate of 0.1 mV s^−1^ in voltage range 0.01–3 V as shown in **Figure** **5**a. Possible electrochemical oxidation reduction reactions of Sn, SnO*_x_*, and carbon in C@1000 °C, C–SnO*_x_*@1000 °C, and CO–SnO*_x_*@1000 °C are mentioned in Equations (1)–(3), 2, 5, 7, 8

(1) $$8.4{\mathrm{Li}}^{+}\;\;+\;\;{\mathrm{SnO}}_{2}\;\;+\;\;8.4e^{-}\;\;=\;\;{\mathrm{SnLi}}_{4.4}\;\;+\;\;2{\mathrm{Li}}_{2}O$$

(2) $$x{\mathrm{Li}}^{+}\;\;+\;\;\mathrm{Sn}\;\;+\;\;xe^{-}\;\;↔\;\;{\mathrm{Li}}_{x}\mathrm{Sn}\left(0\;\;\leq \;\;x\;\;\leq \;\;4.4\right)$$

(3) $$y{\mathrm{Li}}^{+}\;\;+\;\;\mathrm{Carbon}\;\;+\;\;ye^{-}\;\;↔\;\;{\mathrm{Li}}_{y}C$$

From CV as shown in Figure 5a, delithiation of C–SnO*_x_*@1000 °C at 0.1 mV s^−1^ scan rate presents four distinct oxidation peaks but at higher potentials (0.43, 0.65, 0.73, and 0.8 V). Lithiation of C–SnO*_x_*@1000 °C presents three reversible peaks at 0.34, 0.5, and 0.62 V, which are associated with lithiation reactions between Sn and Li. A broad peak centered at 1.2 V (starting at 1.5 V and ending at 0.7 V) can be observed in first lithiation, but disappears in second cycle. This peak is attributed to the decomposition of electrolyte to form SEI films, resulting in irreversible capacity. A decrease in peak current with increase in number of cycles hints about capacity fading happening in C–SnO*_x_*@1000 °C due to irreversible capacity loss during stable SEI layer formation.^13^, ^14^, ^15^

Figure 5a shows five oxidation peaks for CO–SnO*_x_*@1000 °C sample at 0.12, 0.17, 0.23, 0.48, and 1.28 V. First three oxidation peaks 0.12, 0.17, and 0.23 V are due to lithium extraction from carbon matrix in electrode. Oxidation peak at 1.12 V is from delithiation of SnO*_x_* and weak oxidation peak at 1.128 V is due to partly reversible reaction of formation of SnO_2_. Overlap of CV curves for 2nd and 3rd cycles for SnO*_x_* oxidation and reduction confirms reversibility and cycling stability of SnO*_x_* particles in CO–SnO*_x_*@1000 °C.^10^, ^11^

As clearly seen in CV for C@1000 °C in Figure 5a, CV curves overlap well, which confirms that lithium can intercalate and de‐intercalate reversibly in carbon matrix. A broad reduction peak at 0.9 V is due to decomposition of electrolyte to form stable SEI layer. Characteristic CV curves show lithiation happening in carbon close to 0 V versus Li/Li^+^ and delithiation at 0.15, 0.19, and 0.25 V versus Li/Li^+^.^21^, ^22^, ^23^ Figure 5b representing the characteristic charge–discharge versus voltage profile for CO–SnO*_x_*@1000 °C shows the cross over potential of charge/discharge profile at 0.5 V. Capacity drop from 780 to 350 mAh g^−1^ is observed at 100 mA g^−1^ current density. This is attributed to irreversible consumption of lithium ions for stable SEI layer formation.

**Figure 6:**
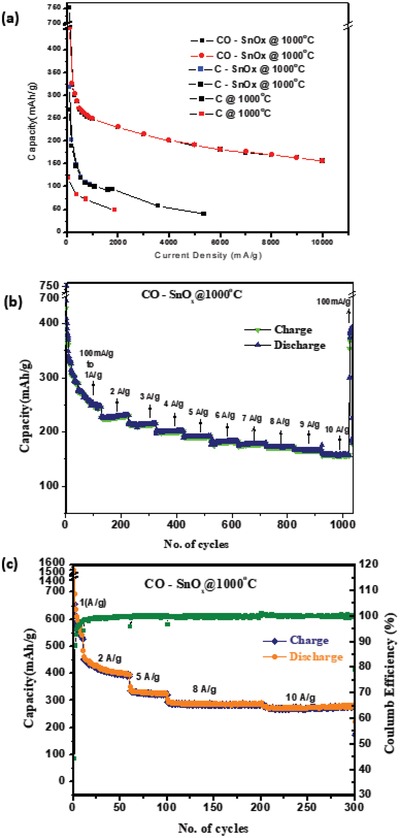
a) Electrochemical performance comparison of C–SnO*_x_* composites versus C@1000 °C. b) Cycling stability and rate capability of CO–SnO*_x_*@1000 °C with starting current density of 100 mA g^−1^. c) Cycling stability and rate capability of CO–SnO*_x_*@1000 °C with starting current density of 1 A g^−1^.

Electrochemical charge–discharge performance of carbon–SnO*_x_* composites carbonized at 1000 °C in the presence and absence of oxygen is compared with carbon sample C@1000 °C prepared from wood fiber as shown in **Figure** **6**a. Charge–discharge capacity obtained for carbon sample C@1000 °C is lower than C–SnO*_x_* samples. This is due to inherent property of carbon and irreversible capacity loss during electrochemical lithiation/delithiation. It is clearly evident from *I*_d_/*I*_g_ ratio listed in Table S1 in the Supporting Information, calculated from Raman spectra that disordered carbon content is prominently high, also graphitic crystallites of carbon have started to appear. Such high amount of disordered carbon content presents unsatisfied carbon bonds, which causes irreversible capacity loss.^31^

Capacity of C–SnO*_x_*@1000 °C has improved slightly when compared with C@1000 °C (Figure 5a). This is obviously due to the presence of SnO*_x_* in matrix. C–SnO*_x_*@1000 °C sample also suffered severe capacity fading and poor rate capability. XRD pattern of C–SnO*_x_*@1000 °C in Figure 2a confirms the presence of tin particles formed during carbothermal reduction. Tin nanoparticle due to its electrochemical alloying mechanism of lithiation/delithiation presents an unstable SEI layer for charge/discharge during each cycle. This SEI layer gets crumbled during volume expansion and every cycle during charge/discharge consumes lithium ions irreversibly. Hence results in irreversible capacity loss and capacity fading. As per literature reports, carbon acts as a mechanical buffer to accommodate volume change. But in this case, in spite of the presence of carbon along with tin nanoparticles, irreversible capacity loss and poor rate capability are observed. This confirms electrochemically unstable nature of tin nanoparticles present in the matrix.^13^, ^16^ As discussed in XPS and FESEM results shown in Figures 3a and 4c respectively, most of the tin nanoparticles available on surface also contribute to irreversible capacity loss and poor rate capability. CO–SnO*_x_*@1000 °C composite sample is a blend of small amounts nanocrystallites of Sn, SnO, and SnO_2_ embedded in carbon matrix. With heat treatment crystallinity of carbon has improved, oxidative etching of disordered carbon is accompanied with partial oxidation of tin. SnO and SnO_2_ are electrochemical conversion family of compounds. They form a stable SEI layer with lithium oxide formed as a by‐product during charge/discharge. Improved stability of SEI layer in combination with increased porosity in this sample makes it an ideal candidate for anode materials in lithium‐ion batteries. Rate capability from 0.1 to 10 A g^−1^ is shown in Figure 6b. Excellent cycling stability after 500 cycles with capacity of 180 mAh g^−1^ at current density of 10 A g^−1^ is obtained in CO–SnO*_x_*@1000 °C sample with initial current density of 0.1 A g^−1^ as shown in Figure S4 in the Supporting Information. To understand the influence of initial current density in obtaining stable SEI layer and to reduce irreversible capacity loss, rate capability electrochemical charge/discharge studies with high initial current density of 1 A g^−1^ is done as shown in Figure 6c. This sample suffered capacity fading initially at 1 A g^−1^ for 10 cycles, 2 A g^−1^ for 40 cycles, and 5 A g^−1^ for 40 cycles. But slowly got stabilized at higher current densities of 8 and 10 A g^−1^ for 100 cycles each. Also extremely stable high capacities of 320 and 280 mAh g^−1^ along with excellent Coulombic efficiency are obtained at higher current densities of 8 and 10 A g^−1^, respectively, as shown in Figure 6c. Outstanding electrochemical performance of CO–SnO*_x_*@1000 °C over other two samples probed for TEM analysis. TEM images of CO–SnO*_x_*@1000 °C in Figure 3d–f confirm the presence of 20–30 nm SnO*_x_* particles dispersed in wood fiber‐derived carbon matrix. Also co‐presence of metallic Sn along with SnO and SnO_2_ is also confirmed as shown in Figure 3e,f. 20–30 nm SnO*_x_* particles embedded in tubular wood fibers are observed. This is in compliance with XRD and XPS results discussed earlier. This structure enables SnO*_x_* particles to breathe lithium ions comfortably during electrochemical lithiation/delithiation. Carbon matrix acts as an effective mechanical buffer to accommodate volume change, strong interface with SnO*_x_* assures continuous electrochemical contact.^17^, ^18^, ^19^, ^29^ To reveal reason for excellent electrochemical behavior of C–SnO*_x_* composites, electrochemical impedance spectroscopy is carried out over a frequency range of 100 kHz to 0.01 Hz as shown in Figure S5 in the Supporting Information along with equivalent circuit for impedance analysis in inset. Judging from diameters of semicircles and referring to randles circuit, bare vertically aligned CO–SnO*_x_*@1000 °C electrode shows smallest charge transfer resistance as compared to those of C@1000 °C and C–SnO*_x_*@1000 °C as shown in Table S3 in the Supporting Information. Semicircle is not closed for C–SnO*_x_*@1000 °C indicating high double layer capacities. Increased phase shift angle (>45°) in case of CO–SnO*_x_*@1000 °C shows that this electrode behaves more like a capacitor with a reduced contribution of solid‐state diffusion as compared to C@1000 °C and C–SnO*_x_*@1000 °C. In CO–SnO*_x_*@1000 °C, electrode structure enables quick transportation of both electrons and ions. Mesoscale architecture is favorable for providing easy access for electrolyte to structure and short diffusion lengths of Li^+^ within material.^13^, ^14^, ^15^

Electrochemical alloying and electrochemical conversion compounds follow slow kinetics of electrochemical lithiation/delithiation. Initially, starting at lower current densities allow long duration exposure of active anode material to form stable SEI layer that results in severe irreversible capacity loss. This fact is overridden with starting current density at 1A g^−1^. There are only very few reports on carbon–tin composites with cycling stability studies for more than 100 cycles at high C‐rates. Achieving high C‐rate capability with no compromise on capacity is a challenge that is attained in this work with just 16 wt% SnO*_x_* content as shown in Table S4 in the Supporting Information. Thus, wood fiber anchored with SnO*_x_* particles displayed excellent C‐rate capability and cycling stability. This is the best result reported so far especially with biomass‐derived carbon composites. Also surface functionalization of wood‐derived cellulose with acetylacetone is a definite breakthrough to replicate nanoscale features in nanocasting for bio‐inspired structures.

## 3 Conclusion

C–SnO*_x_* composites derived from wood fibers have been explored as anode material for lithium‐ion batteries. In templating of wood fibers by impregnation with tin isopropoxide acetyl acetone not only delayed hydrolysis of tin isopropoxide but also stabilized wood fiber structure and helped retain its porosity during carbonization. Second, introduction of 5% oxygen during carbonization led to passivation of metallic tin nanoparticles and yielded SnO*_x_* nanoparticle uniformly distributed through carbon matrix. A porous carbon matrix blended with a mixture of Sn, SnO, and SnO_2_ in CO–SnO_x_@1000 °C is main reason behind superior and stable capacity of 180 mAh g^−1^ at 10 A g^−1^ current density with a 90% capacity retention after 500 cycles. In rate capability studies for initial current density of 1 A g^−1^, extremely stable high capacities of 280 and 260 mAh g^−1^ along with excellent Coulombic efficiency and cycle stability for 100 cycles are obtained at higher current densities of 8 and 10 A g^−1^, respectively. This biomass‐derived carbon–SnO*_x_* composite is certainly a promising anode material.

## 4 Experimental Section

*Material Synthesis*: First step in preparing wood‐based C–SnO*_x_* composite was wood maceration. Pine wood block of 5 g was sliced into small pieces roughly of dimension of 10 mm length and 3 mm thickness. Equal volume of 100% acetic acid and 30% of hydrogen peroxide were added into wood slices and allowed to warm at 80 °C for 12 h in a test tube with screw cap without tight sealing to allow vapor exchange. This step leached out hemicellulose, decomposed lignin, and left behind wood fiber matrix. The wood fiber matrix was washed with distilled water four to five times to remove acetic acid and hydrogen peroxide. After washing, wood fibril matrix was stirred vigorously to separate out wood fibers. The solvent exchange of wood fibers was done with ethanol three times to replace absorbed water content completely. 5 mL of acetylacetone was added to wood fibers. In another glass beaker, 1 mL of tin isopropoxide was added into 5 mL of acetylacetone under stirring. This solution was added in stirring condition to wood fibers treated with acetylacetone. After homogenous mixing, tin isopropoxide impregnated wood fibers were dried at 80 °C for 12 h. Completely dried Sn precursor impregnated wood fibers were carbonized at 1000 °C for 1 h in an argon atmosphere with 10 °C min^−1^ ramp rate. The sample was labeled as C–SnO*_x_*@1000 °C. Another sample with passivated Sn nanoparticles was prepared by carbonization in argon atmosphere accompanied with 5% oxygen introduced at onset of 1000 °C to passivate Sn nanoparticles formed by carbothermal reduction and was labeled as CO–SnO*_x_*@1000 °C. For comparison, acetylacetone‐treated and ‐untreated wood fibers were carbonized at 1000 °C for 1 h to obtain bare carbon fibers labeled as C@1000 °C and CWA@1000 °C, respectively.

*Material Characterization*: FESEM images were taken using a Hitachi SU‐70 analytical ultra‐high resolution SEM (Japan). XRD patterns were recorded by Bruker Smart1000 (Bruker AXS Inc., USA) using CuKα radiation. TGA was carried out using a thermogravimetric analyzer (TA Instruments, USA) with a heating rate of 10 °C min^−1^ in air. To know morphology of materials, high‐resolution transmission electron microscopy (HRTEM, Model: Tecnai G‐20, 200 KV) was done. XPS analysis was carried out to analyze surface chemistry (oxidation states and elemental compositions) of materials using ESCA‐Omicron XPS system with Mg‐Kα as excitation source. Raman spectra were recorded in range between 100 and 2000 cm^−1^ at room temperature using a Raman spectrometer (Model: Horiba JobinYvon Lab Ram HR‐800), with an argon ion laser as a source at an excitation wavelength of 514 nm.

*Electrochemical Measurements*: The carbonized samples were mixed with carbon black and polyvinylidene difluoride binder to form slurry at weight ratio of 80:10:10. Electrode was prepared by casting slurry onto copper foil using a doctor blade and drying in a vacuum oven at 100 °C overnight. Thickness of coating was approximately 20 µm. Electrodes for coin cell testing with diameter 11 mm were cut into circular disks. Batteries were fabricated under same conditions using lithium as anode and 1 m LiPF_6_ in a mixture of ethylene carbonate and diethyl carbonate (1:1 by volume) as electrolyte and circular disks cut from glass microfiber membrane were used as separator. Electrochemical performance was tested using an Arbin battery test station (BT2000, Arbin Instruments, USA). Capacity was calculated on the basis of total mass of the carbonized samples. Cyclic voltammograms were obtained at a scan rate of 0.1 mV s^−1^ between 0.01 and 3 V, charge–discharge studies, cycling stability, and rate capability of the carbonized samples were tested at a current density from 0.1 to 10 A g^−1^ in voltage range 0.01–3 V.

## Conflict of Interest

The authors declare no conflict of interest.

## Supporting information

Supporting Information
