# Supplementary material for: Wood‐Derived Carbon Fibers Embedded with SnOx Nanoparticles as Anode Material for Lithium‐Ion Batteries
Source: Glob Chall. 2019 Nov 8;4(1):1900048. doi: 10.1002/gch2.201900048 (PMC6957017; doi:10.1002/gch2.201900048)
Supplement: Supplementary file 1 — Supporting Information [file GCH2-4-1900048-s001.pdf]

Copyright WILEY-VCH Verlag GmbH & Co. KGaA, 69469 Weinheim, Germany, 2019.

Supporting Information

for *Global Challenges,* DOI: 10.1002/gch2.201900048

Wood-Derived Carbon Fibers Embedded with SnO_x Nanoparticles as Anode Material for Lithium-Ion Batteries

*Janardhanan Revathi, Adduru Jyothirmayi, Tata Narasinga Rao, and Atul Suresh Deshpande**

Supporting information

Wood derived carbon fibers embedded with SnO_x nanoparticles as anode material for lithium-ion batteries

*Janardhanan Revathi, Jyothirmayi Adduru, Tata Narasinga Rao, Atul Suresh Deshpande**

* corresponding author

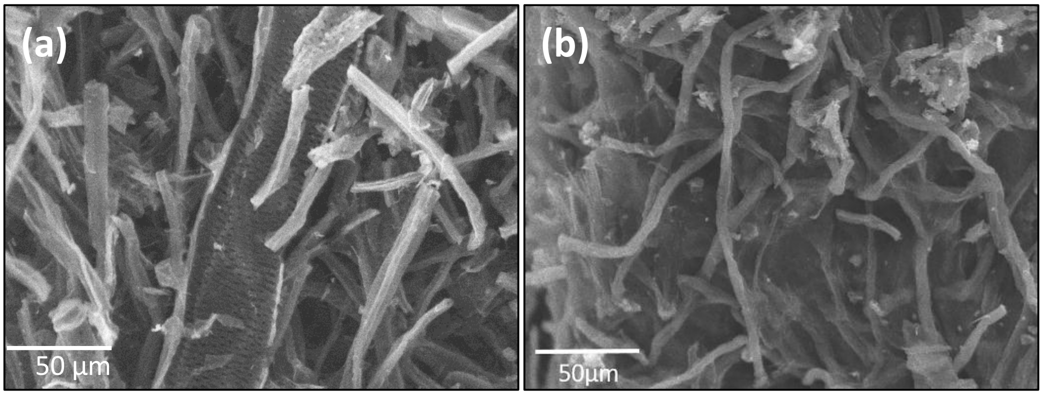

Figure S1. FESEM of (a) C@1000°C and (b) CWA@1000°C

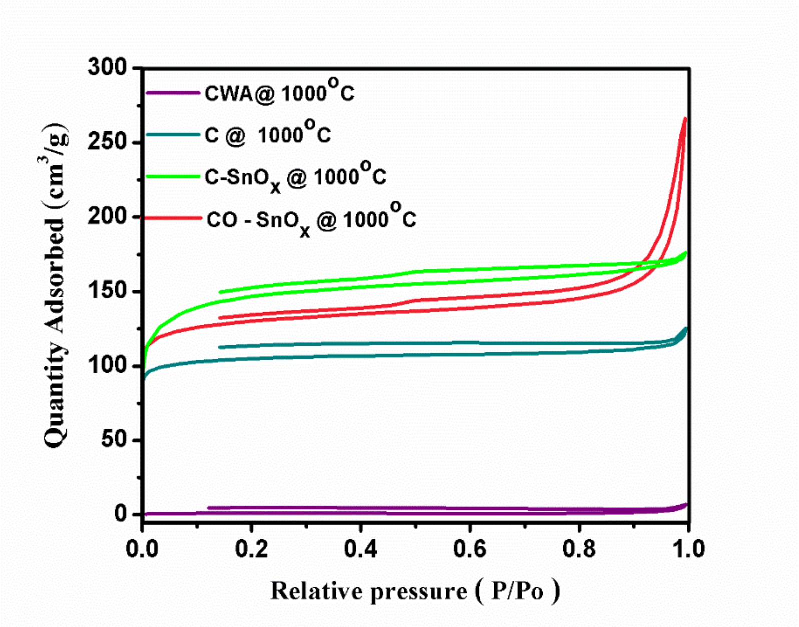

Figure S2. BET isotherm of CO-SnO_x@1000ºC and C@1000ºC

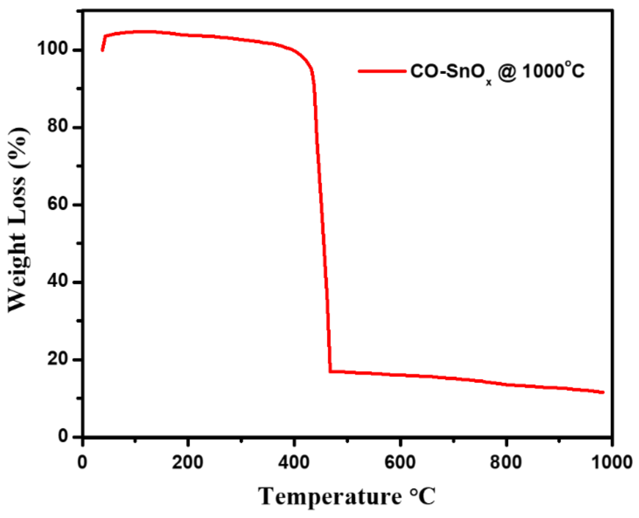

Figure S3. TGA of CO-SnO_x@1000ºC

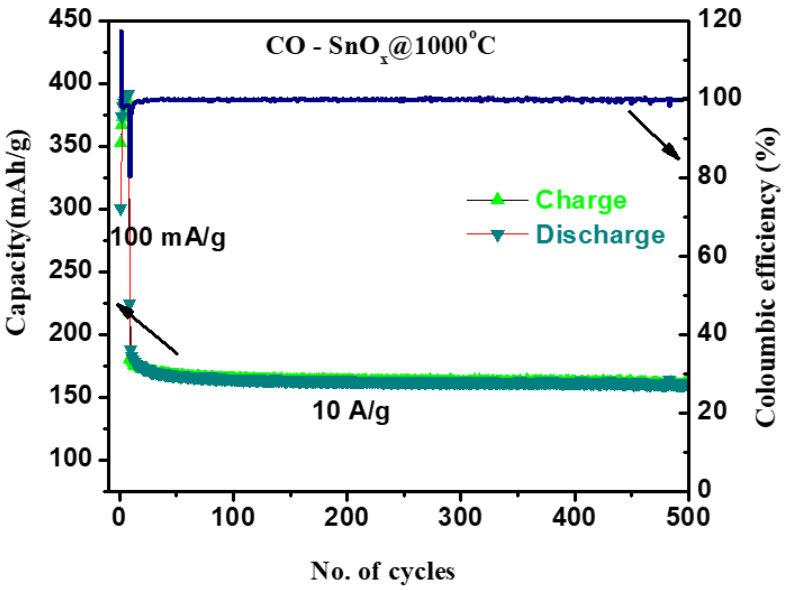

Figure S4. Cycling stability of CO-SnO$_x$@1000°C with starting current density 100mA/g for 5 cycles

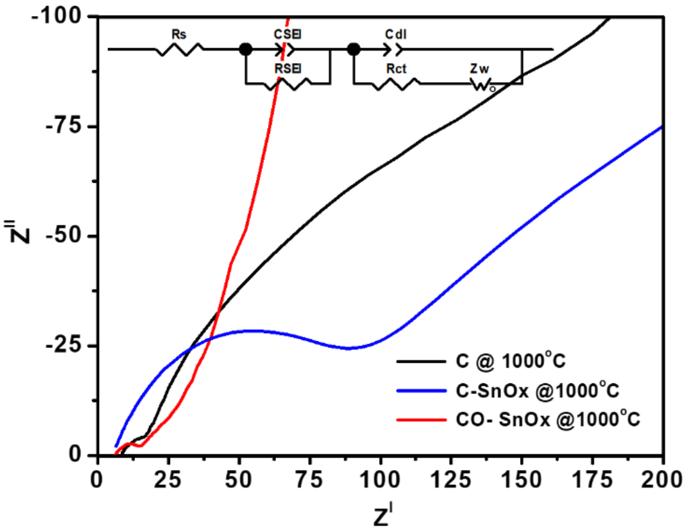

Figure S5. Nyquist plot for C@1000°C, C-SnO$_x$@1000°C and CO-SnO$_x$@1000°C

Supplementary Table 1. I_D/I_G ratio calculated from Raman data for CWA@1000^oC, C@1000^oC, C–SnO_x@1000^oC, CO–SnO_x@1000^oC

**Sample name**	**I_D/I_G***
C	1.75
CWA@1000^oC	1.44
C-SnO_x@1000^oC	1.70
CO-SnO_x@1000^oC	1.66

*** I_D/I_G ratio calculated from integrated area under curve for the D and G bands**

Supplementary Table 2. BET surface area CWA@1000^oC, C@1000^oC, C–SnO_x@1000^oC, CO–SnO_x@1000^oC

**Sample Name**	**BET Surface area m^2/g**	**Average pore diameter (Å)**	**Micropore volume (cm^3/g)**	**Mesopore volume (cm^3/g)**
CWA @ 1000^oC	4	669	0.0112	0.0008
C @ 1000^oC	351	53	0.14	0.05
C – SnO_x @ 1000^oC	499	33	0.16	0.11
CO –SnO_x @ 1000^oC	439	110	0.16	0.25

Supplementary Table 3. Impedance analysis of CWA@1000^oC, C@1000^oC, C–SnO_x@1000^oC, CO–SnO_x@1000^oC

**Impedance analysis parameters**	**C@1000**	**C–SnO_x @1000**	**CO–SnO_x @ 1000**
Rs	8.297	5.189	6.397
RSEI	10.4	77.82	3.956
Rct	4.136	393.5	1.748
Zw– R	1085	47.37	30.52
Zw – T	195.9	3.99	0.254
Zw– P	0.335	0.375	0.476

Supplementary table 4. Literature comparison table

Active material	Sn content	Capacity mAh/g @ current density A/g	No. of cycles	Reference
Carbon-Tin Oxide	16 %	280mAh/g @ 10A/g	150	This work
Porous Carbon-Tin composite	66%	400mAh/g @ 20mA/g<br>300mAh/g @ 1A/g	15<br>15	1
Tin nano particles in elastic carbon matrix	74%	550mAh/g @ 166mA/g	100	2
Tin filled carbon nano tubes	6.33%	800mAh/g @ 37.2mA/g	40	3
SnO_x/Carbon nano hybrids	28%	608mAh/g @ 0.5A/g<br>80mAh/g @ 10A/g	200<br>20	4
Sn-Graphene	46.8%	270mAh/g @ 10 A/g	100	5

Reference

1. Y. Xu, Y. Zhu, Y. Liu, C. Wang, *Adv. Energy Mater.* **2013**, *3*, 128.

2. W. M. Zhang, J. S. Hu, Y. G. Guo, S. F. Zheng, L. S. Zhong, W. G. Song, L. J. Wan, *Adv. Mater.* **2008**, 20, 1160.

3. T. P. Kumar, R. Ramesh, Y. Y. Lin, G. T. Fey, Elec.chem. comm. **2004**, *6*, 520.

4. X. Zhou, Z. Dai, S. Liu, J. Bao, Y. Guo, Adv. Mater. **2014**, 26, 3943.

J. Qin, C. He, N. Zhao, Z. Wang, C. Shi, E. Liu, J. Li, M. Science, ACS Nano, **2014**,
